# The Puzzling Potential of Carbon Nanomaterials: General Properties, Application, and Toxicity

**DOI:** 10.3390/nano10081508

**Published:** 2020-07-31

**Authors:** Danica Jović, Vesna Jaćević, Kamil Kuča, Ivana Borišev, Jasminka Mrdjanovic, Danijela Petrovic, Mariana Seke, Aleksandar Djordjevic

**Affiliations:** 1 Department of Chemistry, Biochemistry and Environmental Protection, Faculty of Sciences, University of Novi Sad, Trg Dositeja Obradovića 3, 21000 Novi Sad, Serbia; danica.jovic@dh.uns.ac.rs (D.J.); ivana.borisev@dh.uns.ac.rs (I.B.); aleksandar.djordjevic@dh.uns.ac.rs (A.D.); 2 Department for Experimental Toxicology and Pharmacology, National Poison Control Centre, Military Medical Academy, Crnotravska 17, 11040 Belgrade, Serbia; v_jacevic@yahoo.com; 3 Department of Pharmacological Science, Medical Faculty of the Military Medical Academy, University of Defence, Crnotravska 17, 11000 Belgrade, Serbia; 4 Department of Chemistry, Faculty of Science, University of Hradec Kralove, Rokitanskeho 62, 50003 Hradec Kralove, Czech Republic; 5 Biomedical Research Center, University Hospital Hradec Kralove, Sokolska 581, 50005 Hradec Kralove, Czech Republic; 6 Oncology Institute of Vojvodina, Faculty of Medicine, University of Novi Sad, Put dr Goldmana 4, 21204 Sremska Kamenica, Serbia; mrdjanovic.jasminka@onk.ns.ac.rs; 7 Department of Natural Sciences and Management in Education, Faculty of Education Sombor, University of Novi Sad, Podgorička 4, 25101 Sombor, Serbia; petrovid@tcd.ie; 8 Institute of Nuclear Sciences “Vinca”, University of Belgrade, Mike Petrovića Alasa 12-14, 11351 Vinča, Belgrade, Serbia; marianaseke@yahoo.com

**Keywords:** carbon nanomaterials, fullerene, nanodiamonds, carbon dots, graphene, carbon nanotubes, toxicity, *in vitro* and *in vivo* research

## Abstract

Being a member of the nanofamily, carbon nanomaterials exhibit specific properties that mostly arise from their small size. They have proved to be very promising for application in the technical and biomedical field. A wide spectrum of use implies the inevitable presence of carbon nanomaterials in the environment, thus potentially endangering their whole nature. Although scientists worldwide have conducted research investigating the impact of these materials, it is evident that there are still significant gaps concerning the knowledge of their mechanisms, as well as the prolonged and chronic exposure and effects. This manuscript summarizes the most prominent representatives of carbon nanomaterial groups, giving a brief review of their general physico-chemical properties, the most common use, and toxicity profiles. Toxicity was presented through genotoxicity and the activation of the cell signaling pathways, both including *in vitro* and *in vivo* models, mechanisms, and the consequential outcomes. Moreover, the acute toxicity of fullerenol, as one of the most commonly investigated members, was briefly presented in the final part of this review. Thinking small can greatly help us improve our lives, but also obliges us to deeply and comprehensively investigate all the possible consequences that could arise from our pure-hearted scientific ambitions and work.

## 1. Introduction

Carbon has been extensively investigated, especially in the form of carbon nanomaterials (CNMs), and it seemed to behave like a magnet for the Nobel Prize (in 1996 ”for the discovery of carbon atoms bound in the form of a ball,”, and in 2010 ”for groundbreaking experiments regarding the two-dimensional material graphene”). In the last four decades, carbon has expanded its family of novel nanomaterials, which now includes fullerenes, carbon dots (CD), nanodiamonds (NDs), nanohorns (CNHs), nanofibers (CNFs), nanotubes (CNTs), graphene, etc. Carbon nanomaterials usually do not consist of chemical individuals, but form complex mixtures of compounds that only slightly differ in molecular weight, structure, isomerism, etc. It is also known that some of these materials possess different properties and structures if they come from different manufacturers or are even from different lots. Hence, it is difficult to assess the properties of entire groups of materials; instead, group representatives are discussed.

**Figure 1:**
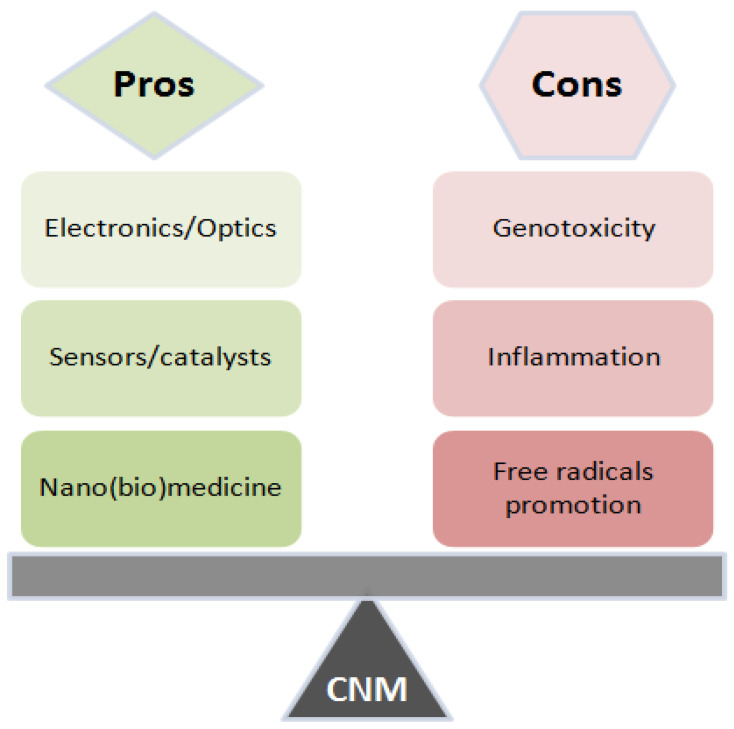
Pros and cons for the carbon nanomaterials (CNMs) use, original figure.

Carbon nanomaterials, as well as their impact, are greatly defined by their physico-chemical properties [1,2,3]. Shape, size, solubility, charge, surface, and chemical functionalization of the nanoparticles are some of the properties that make the system more or less stable and prone to aggregation and self-assembly. By chemical transformation, CNMs can be successfully transformed into more polar or soluble derivatives, thus overcoming one of the greatest obstacles when it comes to the application in biological media. An inevitable consequence of the extensive production and widespread application of nanomaterials is their consequential presence in the environment. Before they are settled in soil or continue to flow within water sources, nanomaterials can be transformed as a result of different processes, thus changing their native properties [4,5]. Accumulation of nanomaterials in the soil and water makes these areas nanointoxicated and, as such, with the yet unravelled potential of causing various effects in plants, animals, fungi, microorganisms, and, consequently, humans. As nanotechnology has bloomed and still has a lot to go until reaching its zenith, the impact of nanomaterials is of utmost importance to be fully addressed. The possible risks [6,7] and toxicity of nanomaterials (Figure 1) have attracted the interest of scientists worldwide. Although a great amount of research has been performed so far, this venture is still in its infancy.

In this review paper, we have selected the most prominent members of the carbon nanomaterial family, briefly summarized their main characteristics and potential application, as well as described their toxicity following the *in vitro* and *in vivo* results.

## 2. General Properties of Carbon Nanomaterials 

Hybridization and bonding between C atoms in carbon nanomaterials are some of the direct guides that classify materials within this group by their properties. For example, all the sp^2^-structured nanomaterials share similar properties such as conductivity, mechanical strength, and reactivity; however, size and shape also make differences among them. For example, C_60_ can be observed as easily soluble, among other, only slightly dispersible, carbon nanomaterials, which usually form unstable dispersions. Besides being sp^2^-structured, there is a group of nanodiamonds that are predominantly sp^3^-structured, and carbon dots that contain various ratios of sp^2^ and sp^3^ carbons [8].

**Figure 2:**
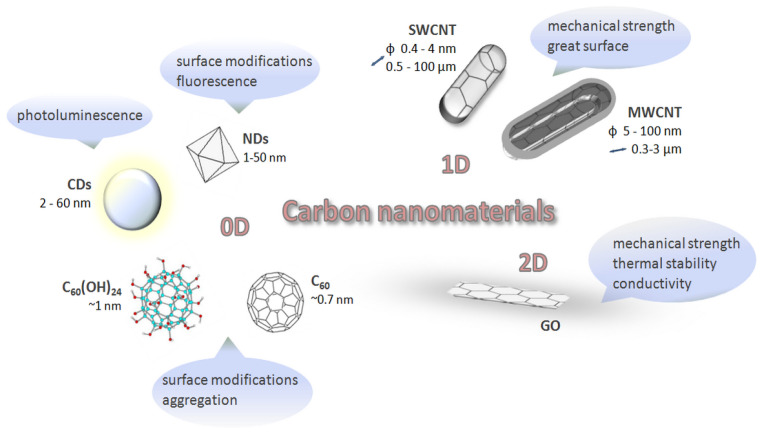
The main physicochemical properties of different nanostructures fullerene (C_60_); fullerenol (C_60_(OH)_24_); carbon dots (CDs); nanodiamonds (NDs); single-walled carbon nanotube (SWCNT); multi-walled carbon nanotube (MWCNT); graphene oxide (GO).

Carbon nanomaterials have so far found their purpose in optics, medical implants, medical electronics, tissue scaffolds, and sensors, as well as in other biomedical devices and as a promising drug/gene/vaccine-delivery system [4,9]. Based on the dimensionality of their structure, carbon nanomaterials can be classified in one of the following groups: Zero-dimensional (0D), one-dimensional (1D), or two-dimensional (2D) materials (Figure 2).

### 2.1. Zero-Dimensional (0D) Carbon Materials

0D materials have all dimensions less than 100 nm, i.e., within the nanoscale range, and this group comprises fullerenes, carbon dots, nanodiamonds, and nano-onions.

#### 2.1.1. Fullerenes

**Figure 3:**
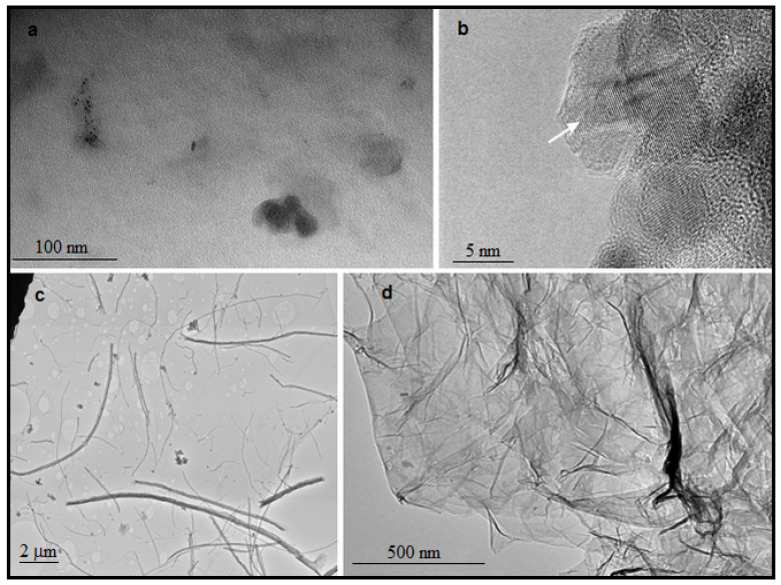
(**a**) TEM/HR-TEM images of different carbon nanomaterials: C_60_ nanoparticles (scale bar = 100 nm), original figure; (**b**) nanodiamonds (scale bar = 5 nm), adapted from [26], (**c**) carbon nanotubes (CNTs) (scale bar = 2 μm), original figure; (**d**) graphene oxide (scale bar = 500 nm), adapted from [27].

Although football is tremendously popular worldwide, it came as a surprise when, in 1985, it also entered the scientific niches in the form of the newly discovered C_60_ fullerene molecule. Fullerenes are closed hydrophobic carbon nanocages, with a size and stability depending on the number of C-atoms. One of the most prominent and studied members of this cluster is fullerene C_60_, and it presents a structure of high symmetry, unique physicochemical properties, extraordinary beauty, and stands in between molecule and nanoparticle, by being both at the same time (Figure 3a). The pyramidalized orientation of carbons, rather than the planar one, causes sp^2^ carbon atoms to take a pseudo-sp^3^ form, thus making this molecule reactive and easy to modify [8].

The wide spectrum of fullerene applications goes from electronics, optics, and cosmetics to catalysts, the environment, and nanomedicine [10,11,12].

One of the obstacles in fullerene manipulation is its poor solubility in water, which was successfully overcome by the functionalization of a molecule, thus obtaining different biocompatible water-soluble derivatives [13,14,15]. The most studied ones are hydroxylated fullerenes, among which fullerenol C_60_(OH)_24_ proved to show quite a broad spectrum of biological activity [16,17,18]. C_60_(OH)_24_ has symmetrically arranged hydroxyl groups attached to the C_60_ cage and reaches a size of approximately 1 nm. One of the characteristics that arise from the structure is aggregation and agglomeration, where the size of aggregates can vary from several to hundreds of nanometers, with the average size of 40–60 nm [19,20,21].

Some of the properties of fullerenol that proved to be very promising are its antioxidant activity [22] and protective effects [23], as well as the potential use in biomedical purposes such as nanodrug delivery [24,25].

#### 2.1.2. Carbon Dots

Carbon dots represent novel zero-dimensional carbon nanostructures [28] that are easily soluble in water and with excellent optical properties, e.g., strong photoluminescence, which is why they are called carbon nanolights [29]. According to the literature data, several divisions of carbon dots can be made based on their internal carbonaceous structure [28,30,31,32]. Their size and shape can vary [32,33,34,35,36,37,38,39,40,41], and one of the most important tunable properties of CDs is the photoluminescence effect, which strongly depends on the carbon dots’ characteristics (type, size, crystallinity, surface properties, etc.) [31]. CDs also exhibit other important tunable properties such as: Phosphorescence, electrochemical luminescence, up-conversion luminescence, tunable emission wavelengths, stable fluorescence, and resistance to bleaching [38,39].

One of the most important tunable properties of CDs is the photoluminescence effect (PL), which strongly depends on the carbon dots’ characteristics (type, size, crystallinity, surface properties—oxidation degree, doped heteroatoms, etc.) [31]. The quantum yield (QY) (QY is the number of emitted photons relative to the number of absorbed photons) of raw carbon quantum dots is low as a result of emissive traps on the surface, and therefore, surface modifications and/or size modulation are required for achieving better brightness [31,32]. Graphene quantum dots have higher quantum yields due to their multilayered structure and more pronounced crystallinity, but all these properties can also be significantly improved by surface modifications in terms of synthetic methods development [31,32,42,43]. The exact mechanism of PL is still not completely clarified, but it has been notified that mainly the PL emission spectra maxima of CDs are located in the blue and green region, while PL emission in the ultraviolet (UV) and visible region is not suitable for *in vivo* imaging, due to the tissue autofluorescence and serious photodamages generated by UV excitation [44], which, on the other hand, can also be overcome by innovations in synthetic procedures for CDs [45]. CDs express immense photostability, which enables long-term real-time imaging and non-blinking photoluminescence, which is an important feature for single-molecule tracking [33,46,47].

The carbon core structure is necessary for bioimaging, the effects of light-emitting diodes, mass spectrometry, and thermal therapy, whilst the nature of functional groups at the surface is responsible for enzyme and gene regulation effects, electro- and organo-catalysis, and drug delivery applications [48,49,50]. As photoluminescence is essential for bioimaging and biosensing applications, to date, enormous efforts are being made with the following final goal: To precisely define factors that enable the accurate and reproducible synthesis of materials with optimal photoluminescence properties [31,48]. The electronic and optical properties of CDs direct their use toward chemical catalysis, electronics, sensors, applications in trace detectors of explosives, food quality control, etc. [30,39,46]. As CDs are able to detect temperature, humidity, and pH, as well as to detect and monitor heavy metals (Hg^2+^, Pb^2+^, Cd^2+^, etc.), their applications as environmental sensors should also be noted [26,37,51,52,53,54,55,56]. Mentioned properties can be achieved through processes of passivation and functionalization of CDs, which further lead to the development of portable devices containing CDs-based heavy metal sensors, which certainly represents a big step toward the new eco-friendly and cost-effective practical use of these nanomaterials [57,58,59].

Besides, CDs have high solubility in aqueous media, a good toxicology profile, and biocompatibility [57,58], and hereof can be used in biomedicine as fluorescent and multimodal bioimaging agents [47,59,60,61], biosensors [28,41,62,63], and targeted drug delivery applications [64]. Systematic representations of achievements in the field of carbon dots-based biosensors used in cancer diagnostics for tumor marker detection, analysis of cancer cells, and bioimaging purposes were summarized in a paper written by Pirsaheb et al. [44].

#### 2.1.3. Nanodiamonds

Although nanodiamonds were discovered in the 1960s, the exact structure had remained undefined for more than 30 years [65].

Depending on the starting material and the purification processes, the sizes of the obtained ND (Figure 3b) particles vary [66,67,68,69,70,71]. Some ND particles are faceted, mainly forming a round shape (3–5 nm in diameter), whereby every particular nanodiamond is composed of a diamond core [68,72]. Water dispersions of NDs obtained by the ozone purification method are stable in a wide pH range (2–12) and have zeta (ζ) potential values from −50 to −100 mV, depending on the polydispersity of the system [69,70,71].

The possibility of various surface modifications is one of the most beneficial features of NDs. This can be achieved by attaching different functional groups to their surface without interrupting the essentially important structure of a highly ordered diamond core [73,74], or by the doping of NDs. The surface modification step is of high importance for biomedical applications as it allows the prediction of possible interactions of NDs with biological surroundings in the living systems by designing NDs with high dispersibility, good solubility, and stability in biological pH conditions [75].

Fluorescence, as the fundamental property of NDs, is based on advantages such as small sizes, high photostability, multicolor fluorescence, diverse surface chemistry, and good toxicology profile, making NDs an immensely promising candidate for advanced *in vivo* imaging [76,77]. NDs are used in electronics, as light and electron-emitters; in spintronics; in electrochemistry, as bright, low-voltage (cold) cathodes; and in the biomedical area, as drug delivery, macromolecules delivery, bioimaging, etc. [10,66].

### 2.2. One-Dimensional Carbon Nanomaterials

1D materials have two dimensions within the nanoscale, i.e., materials whose only one dimension is above 100 nm, and this group comprises carbon nanotubes, carbon nanofibers, and carbon nanohorns.

#### 2.2.1. Carbon Nanotubes

Carbon nanotubes are made of carbon hexagons arranged in sheets that form cylindrical structures. CNTs can exist as single-walled (SWCNTs) with diameters of 0.4–2 nm, or multi-walled (MWCNTs) carbon nanotubes that can have diameters up to 100 nm. The length of CNTs, however, can reach the nano or microscale, as can be seen in Figure 3c. Despite a plethora of syntheses and separation processes, obtaining uniform CNTs is still a challenge [78]. Great surfaces and hollows make CNTs a good candidate for the delivery of drugs and macromolecules, as well as for photodynamic therapy, and as a contrast therapeutic agent [79]. Being hydrophobic, CNTs are insoluble in biological media, which implies the risk of cytotoxicity. Both non-covalent and covalent functionalization [80,81] allow CNTs to be applied in biomedical purposes. Covalent functionalization is mostly performed with amino groups, by oxidation, hydrogenation, thiolation, and radical reactions [82,83,84]. On the other hand, non-covalent interactions are established between CNTs and biologically active molecules, but with the preserved intact chemical structure of the active principle. Proteins and nucleic acids are subject to this kind of functionalization [85], but some studies included non-covalent interactions between CNTs and drugs [86]. Owing to their unique structure, and mechanical and electronic properties, CNTs have an important role in the design and development of biosensors and biocatalysts [87].

#### 2.2.2. Carbon Nanohorns

The structure of nanohorns is quite similar to one of the CNTs [88], but with a more preserved structure as a result of the synthesis pathway and separation process. Their structure includes closed graphitic tubes with conical buds and with a diameter within 2–5 nm and length of 40–50 nm. CNHs are chemically stable with a great specific surface, high purity, and low toxicity. They exhibit good catalytic properties and conductivity. Ions of metals such as scandium, yttrium, lanthanum, and gadolinium could, during synthesis processes, successfully be intercalated within the holes in the structure, which enables nanohorns to be applied as a diagnostic agent in magnetic resonance imaging [89].

### 2.3. Two-Dimensional Carbon Materials

2D carbon nanomaterials have only one dimension within the nanoscale range and the most prominent example of this group is graphene, as well as its derivatives: Graphene oxide (GO), reduced graphene oxide (rGO), graphene nanoribbons, and graphitic multilayered nanosheets [8,90,91].

#### Graphene

Graphene was discovered in 2004 [92], and it presents an atom-tick sp^2^ carbon lattice in which carbons follow the hexagonal pattern. It has been developed and modified in different shapes, sizes, and derivatives [93]. For its outstanding mechanical strength, graphene is considered to be Popeye among the materials. Moreover, high electronic conductivity and good thermal stability make graphene an excellent material for technological application in electronics, energetics, mechanics, catalysis, and sensorics [94]. Besides, graphene and its derivatives also proved to be very potent and promising for biomedical purposes, where biosensing, bioimaging, drug delivery, bioassays, and tissue scaffolds [95] seem to be the most studied ones.

Chemical modifications of graphene offer more compatible and potent forms that, by functionalization, change the physico-chemical properties as well. An economic way to obtain graphene-like material is the synthesis of GO from graphite, which is recognized as cost-effective and generally safe [91]. Different pathways result in a product of different material quality and size, cost-effectiveness, as well as scalability [91,96].

GO (Figure 3d) is a result of exfoliation of graphite oxide that is synthesized after graphite΄s exposure to strong oxidizing agents, and one of the widely used syntheses is Hummer’s method [91,97]. After this step, ultrasonication enables extraction of GO sheets. For the exceptional mechanical, thermal, and electrical characteristics, GO has to thank its structure that combines sp^2^- and sp^3^-hybridized carbons, while oxygen-containing functional groups make the material hydrophilic and also allow hydrogen bonding [98]. rGO is being synthesized as a product of *GO* reduction by a treatment that can be chemical, thermal, or microwave-assisted [98,99]. Reduced graphene oxide has quite similar thermal and mechanical properties to graphene, thus enabling *rGO* to be widely used for the electronics.

## 3. Biological Effects of Carbon Nanomaterials

Some excellent properties of carbon nanoparticles (CNPs) result in their wide application in different fields including biomedicine and cosmetology. For instance, CNPs might be used for drug delivery, regenerative medicine, and cancer diagnosis and therapy [100]. However, their unique properties might also cause adverse health effects. Consequently, as DNA damage, caused by CNPs, may initiate and promote carcinogenesis, impact fertility, or contribute to ecogenotoxicity, a genotoxicological evaluation turns out to represent a vital area considering the health risk assessment. No less important is the fact that evaluation of the carcinogenic or mutagenic potential of new substances, and thus CNPs, is an important part of preclinical safety testing of novel pharmaceuticals, which is a necessity before moving on to Phase I/II clinical trials. In general, knowledge about the genotoxicity of the CNPs allows us to understand and then minimize the potential adverse effects associated with them, in order to protect human health and the environment.

### 3.1. Genotoxic Preview of Carbon Nanomaterials

The genotoxicity of carbon nanoparticles (CNPs) is predominantly related to their contact with cellular macromolecules, especially DNA. Nanoparticles (NPs) that cross the cell membrane can enter into the nucleus by diffusion through a nuclear membrane or nuclear pores and react with the DNA. Smaller NPs (8–10 nm in diameter) can enter the nucleus via nuclear pores, whereas larger NPs (15–60 nm in diameter) may reach out to the DNA during cell division when the nuclear membrane dissolves [101]. Large NP aggregates can even induce the formation of cellular vesicles and consequent deformation of nuclear shape, which negatively affects the process of mitosis. This leads to physical interference with correct segregation of chromosomes, and the functioning of the mitotic spindle and its components. Such NP aggregates could also mechanically damage the chromosomes [102]. The final outcome of the effects of CNPs to DNA is determined by a range of physico-chemical properties of NPs such as size, shape, surface properties, composition, solubility, aggregation/agglomeration, uptake, and presence of mutagens and transition metals from the NP surface. Comprehensive analysis of different aspects of CNP genotoxicity is essential as, besides the acute genotoxic effects of NPs, long-term exposure can induce mutation.

#### 3.1.1. The Genotoxicity of Fullerene

A range of *in vitro* and *in vivo* studies have demonstrated the absence or minimal genotoxic effects induced by fullerenes and their derivatives [103,104,105,106,107]. The observed genotoxicity of fullerenes and their derivatives is usually caused by photo-induced DNA damage by interacting with NADH and the consequent generation of reactive oxygen species (ROS) [107]. It should be emphasized that even though fullerene possesses antioxidant capacity, which has made it a promising core ingredient in many skincare products, it has the potential to display a range of activities resulting in cell death or dysfunction. In addition, chronic effects of fullerene exposure are still unclear and, thus, this aspect deserves particular attention (Table 1).

#### 3.1.2. The Genotoxicity of Nanodiamonds

Despite the increasing importance of NDs, the data on their toxicity are still limited and conflicting (Table 1). Multiple options for surface modification of nanodiamonds, as well as their carbon-based nature, emerge, the attention focusing toward their toxicity. It has been proposed from *in vivo* studies that induction of oxidative stress that may accompany long-term exposure to NDs is responsible, at least in part, for their toxicity [108,109]. For example, Dworak and coworkers demonstrated that NDs-mediated oxidative stress may contribute to DNA damage on lymphocytes, which is susceptible to prolonged treatment to NDs [110]. Anyway, a systemic investigation of long-term toxicity is needed to reach clinical trials.

#### 3.1.3. The Genotoxicity of Carbon Nanotubes

Various mechanisms have been proposed for the genotoxicity of CNTs, including direct interactions with genomic materials, direct and/or indirect ROS generation, interference with the centrosome and spindle apparatus, and disrupting mitochondria (Table 1). Moreover, transition metal residues remaining after CNT synthesis participate in Fenton-like reactions and induce higher ROS production than pure CNTs. Donaldson and coworkers reported that CNTs can induce additional inflammation or ROS production, suggesting secondary genotoxicity [119]. CNTs can also act as an ROS-scavenging and quenching agent. Proper organic surface functional groups and structural defects can donate electrons to reactive radicals and neutralize their effects. The mode of action of this double-edged sword is material-, solution-, impurity-, and assay-dependent [107].

#### 3.1.4. The Genotoxicity of Graphene

Various *in vivo* studies have assessed the genotoxic potential of graphene and its derivatives, and the results vary from the complete absence of genotoxicity to confirmed DNA damage [117,118,120] (Table 1).

It was revealed that the small lateral diameter of graphene can directly interact with DNA and induce genotoxicity through ROS production and oxidative DNA damage. However, in many cases, it is not exactly clear whether the produced ROS originated directly from the graphene surface or indirectly from cellular sources, such as mitochondria and leukocyte inflammation [107]. Anyway, the size, surface morphology, surface functionalization, and platelet structure of graphene and its derivatives determine their effects, that is, direct or indirect mechanism of genotoxicity (Table 1).

### 3.2. Mechanisms of CNP-Induced Genotoxicity

When speaking of genotoxicity of NPs, and thus, CNP, it is still unclear whether its effect on DNA is nanospecific or not. Primary mechanisms of the genotoxicity of NPs include direct and indirect ones.

*Direct genotoxicity* from the physical interactions of NPs with DNA [121]. Depending on the cell cycle stage, the NPs that entered the nucleus may react either directly with DNA organized in chromatin or with chromosomes. During the interphase, NPs can interact or bind to DNA and mechanically inhibit the replication and interfere with transcription processes. For example, CNP in *Escherichia coli* most likely binds to single-stranded DNA during replication, incorporates into the DNA duplex, and consequently inhibits bacterial growth [122]. NPs may also directly react with chromosomes during mitosis, which leads to chromosome breakage and disturbance of mitosis, thus causing consequent clastogenic or aneugenic effects.

*Indirect genotoxicity* can result from reduced DNA repair function or increased production of ROS upon interaction with other cellular components (e.g., mitochondria, cell membrane), resulting in antioxidant depletion and altered gene expression. NPs are considered responsible for non-specific oxidative damage, which is the predominant cause of DNA damage and subsequent genotoxicity. Indirect genotoxicity can result from:

- *NP interactions with nuclear proteins implicated in replication, transcription, or repair processes:* For example, C_60_ fullerene binds to DNA topoisomerase II alpha in the ATP binding domain, which may inhibit enzyme activity [123]. C_60_ fullerene might interact with PMS2, RFC3, and PCNA proteins involved in the DNA mismatch repair pathway [124]. Moreover, NPs can induce ROS-mediated inactivation of nuclear proteins, thus causing structural alteration thereof [125].
- *NP interactions with a mitotic spindle or its components-aneugenic effect:* NPs interreacting with the mitotic spindle apparatus, centrioles, or their associated proteins can affect any of the mitotic apparatus functions, which can eventually lead to a loss or gain in chromosomes in daughter cells. This interpretation is supported by the results obtained by Sargent et al. [114], who reported the induction of aneuploidy, the formation of three spindle poles and microtubules, and centrosome fragmentation in human airway epithelial cells exposed to SWCNTs at a dose that would be a worker-relevant exposure dose (0.024 µg/cm^2^). Similar effects were observed for MWCNTs on the same cell type [115].
- *Disturbance of cell cycle checkpoint functions:* NPs can react with protein kinases and affect their function. It is well-established that protein kinases are responsible for cell cycle regulation, i.e., replication of DNA and cell division. Inactivation of protein kinases or NPs interaction with proteins involved in the aforementioned processes can result in the disturbance of protein kinase function. Such disturbance of cytokinesis can consequently lead to the formation of aneuploid or multinucleated cells [116].
- *ROS arising from NP surface:* NPs can cause ROS in the cells that may, through free radical attack, generate indirect oxidative damage to DNA. Namely, ROS attack the DNA, causing purine- (such as 8-oxoG) and pyrimidine-derived oxidized base lesions and DNA strand breaks. Such damages of the DNA base can cause mutations through mispairing in replication, leading to carcinogenesis [126].
- *Transition metals that form the NP surface, as a consequence of the synthesis pathways*: DNA damage can be generated by toxic ions released from soluble NPs.
- *ROS produced by cell components (mitochondria):* DNA damage can be caused by ROS, which occurs as a mitochondrial response to stress produced as a result of NP cell components interactions.
- *Inhibition of antioxidants defense:* The inhibition of antioxidants and consequent accumulation of reactive oxygen can potentially lead to DNA damage [125].

Contrary to indirect genotoxicity, secondary genotoxicity can be a result of oxidative DNA attack by ROS generated by activated phagocytes (neutrophils, macrophages) during NP-induced inflammation [127]. Inflammation is most commonly associated with genotoxicity.

### 3.3. Activation of Cell Signaling Pathways by Carbon Nanoparticles

CNPs are predominantly synthetic structures, and the main question arises whether the interactions between them and cells are similar or essentially different from already known interactions between cells and their physiological ligands [128]. Once the CNP comes into contact with the cell membrane, they activate a sequence of signal transduction cascading events, resulting in various cellular responses, such as cell death, cell growth, survival, migration, communication, differentiation, proliferation, autophagy, excretion, and changes in cell metabolism (Table 2).

Cells communicate with its environment via transmembrane receptors that bind ligands (extracellular signaling molecules) and, through the chain of chemical messengers inside the cell, receptors amplify the signal from the environment and convert it into responses strong enough to be passed on to the nucleus or other sites within the cell.

**Figure 4:**
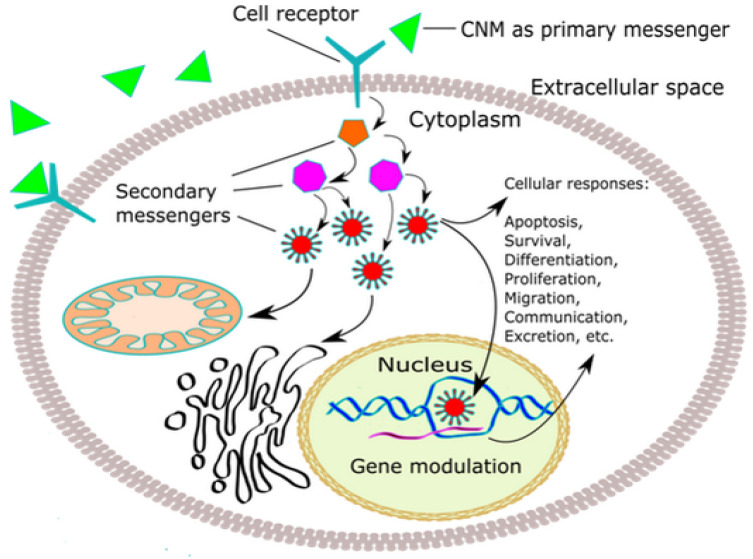
General scheme of cell signaling cascade, original figure.

Transmembrane receptors obtained their names by a mechanism via which they transduce the signal and can be classified into three main categories: G-protein-coupled receptors [148], ion channel receptors [149], and enzyme-linked receptors [150]. Some receptors are placed inside the cell in different compartments such as cytosol [151], nucleus [152], and mitochondria [153]. These receptors bind signaling molecules that pass through the plasma membrane freely, by the process of diffusion, or through the process of endocytosis, which can be clathrin-dependent and clathrin-independent [154]. Considering CNPs, it is very difficult to predict the exact mechanism of CNP internalization and subsequent cellular localization due to their great variety in charge, size, morphology, functional groups, and stability. As CNPs are an innumerable group of nanomaterials, the present review deals with the activation of intracellular signaling pathways activated by the specific CNPs (Figure 4).

#### 3.3.1. Graphene

Interaction of CNPs with membrane receptors means that they can actively and specifically modulate signal transduction pathways [155]. In the research of Chen and coworkers, graphene oxide nanosheets induced autophagy of macrophages through interaction with toll-like receptor (TLR) signaling cascades, including TLR4 and TLR9 with their downstream signaling mediators such as MyD88, TRAF6, and nuclear factor (NF)-κB. This played a crucial role in triggering cytokine inflammatory responses [129]. Oxidized graphene can affect other transmembrane receptors as well. Thus, oxidized graphene nanoribbons that are non-covalently functionalized with PEG-DSPE (1,2-distearoyl-sn-glycero-3-phosphoethanolamine-N[amino(polyethyleneglycol)])(O-GNR-PEG-DSPE) are able to activate epidermal growth factor receptors (EGFRs). This interaction initiates a dynamin-dependent macropinocytosis-like response where the outcome is significant O-GNR-PEG-DSPE uptake into cells with high EGFR expression [130]. EGFR is an enzyme-linked receptor with kinase activity that is important for the induction of apoptosis and cell proliferation [156]. When ultrafine CNPs (Printex 90) were applied on the rat lung epithelial cells, they induced cell proliferation by activating transmembrane receptors, EGFR and β1-integrin [131]. Integrin receptors play a key role in mediating cell adhesion and communication between cells. Their activation by extracellular ligands stimulates processes such as angiogenesis, differentiation, and migration [132], as well as suppression of anoikis [157] through protein kinase B (Akt) mediation [133]. In addition, β1-integrin is capable of promoting fibrosis through activation of downstream proteins: P21-activated kinase and the Yes-associated protein 1 [158].

#### 3.3.2. Fullerenols

Oxidative stress, which includes the production of ROS and NO molecules, results in the downstream induction of the transcriptional factor NF-kb, proinflammatory cytokines (tumour necrosis factor (TNF)-α, interleukine (IL)-1β, or IFN-γ), and cellular kinases associated with the promotion of cell proliferation [159,160]. In this regard, more than two decades ago, it has been suggested that fullerenol-1 (with 13–15 hydroxyl substituents) exhibits inhibitory effects on signal transduction pathways, where fullerenol-1 mediated the antiproliferative effect on vascular smooth muscle cells through the inhibition of the membranous protein tyrosine kinase [134]. This antiproliferative effect of fullerenol-1 is of great importance because it prevents the abnormal accumulation of vascular smooth muscle cells, inflammatory cells, and extracellular matrix proteins, which are altogether the characteristics of atherosclerosis [161]. Since that time, molecular biological mechanisms of the antioxidative activity of fullerenols were not quite yet resolved, but some details are. It is known that the key role has transcriptional factor Nrf2, which regulates the expression of a couple of genes involved in antioxidative defense [135]. The regulation of gene expression involves the binding of Nrf2 to their antioxidative elements on DNA, modulating the activity of antioxidative genes. In physiological conditions, Nrf2 is bound to the receptor Keap 1 in the cytoplasm, where it undergoes degradation by the ubiquitin-proteasome system [162]. Following the activation, Nrf2 is released from the Keap1 protein and translocates into the nucleus where it forms a heterodimer with the Maf protein and goes into the transcriptional machinery responsible for antioxidative defense. Fullerenol C_60_(OH)_24_ induces the translocation of Nrf2 into the nucleus of human lung cells and enhances the expression of antioxidative enzymes such as: Heme oxygenase-1, NAD (P) H: Quinone oxidoreductase 1 and γ-glutamate cysteine ligase [135]. Furthermore, fullerenol C_60_(OH)_24_ is involved in phosphorylation and activation of p38 mitogen-activated protein kinase (MAPK) kinase, and then the kinases, which are regulated by extracellular signals, as well as c-Jun-N-terminal kinases [135]. All mentioned kinases phosphorylate Nrf2 and enable its translocation into the cell nucleus [163,164].

#### 3.3.3. Carbon Dots

Several studies have reported the very low cytotoxicity of CDs toward the human hepatocellular carcinoma Hep G2 cells [165], as well as human kidney embryonic 293T cells [166,167], human breast cancer cell line SKBR3, and normal human breast epithelial cells (MCF-12A) [168]. The absence of toxic effects in the cells is possibly due to the significantly smaller size of C dots, its higher hydrosolubility, and a greater degree of oxidation [165]. However, C dots were found to, in a dose-dependent manner, generate a ROS response in yeast cells, and this was further enhanced after light exposure [143]. A study by Havrdova et al. [58] demonstrated that, depending on the surface modification, CDs exhibited different levels of toxicity in mouse fibroblasts. Data obtained from PEGylated and positively charged CDs revealed no cytotoxic effects, while data derived from pristine CDs (negatively charged) showed the excessive generation of ROS and arrest of the G2/M phase of the cell cycle. Additionally, positively charged polyethyleneimine-coated CDs displayed the highest toxicity with significant changes in the G0/G1 phase of the cell cycle [58]. Similarly, carbon dots functionalized with PEG_1500N_ and injected into mice revealed the absence of significant toxic effects *in vivo* up to 28 days [59], while polyethylenimine-coated CDs were toxic to HT-29 cells, with the observation that the increase in number of ethylenimine units is associated with increased cytotoxicity [169]. A study by Qian et al. suggested that certain CDs, such as pristine graphene quantum dots (GQD), as well as 1,2-ethylenediamine (EDA)-functionalized GQDs, exhibited very low toxicity to human HeLa cells. When applied at the concentration lower than 125 mg/mL, cell viability was higher than 80%, indicating that GQD functional modification with EDA organic molecules has had little influence on the cell toxicity [170]. Moreover, modification of the cisplatin(IV)prodrug-loaded CDs with an anionic polymer PEG-polydimethylmaleic acid (CDs-Pt(IV)@PEG-(PAH/DMMA)) has yielded charge-convertible CDs, which, with *in vitro* malignancy settings, demonstrated better therapeutic potency, while the *in vivo* xenograft tumor-bearing mice model exhibited higher tumor inhibition with reduced systemic toxicity [171]. In a very mild acidic tumor extracellular microenvironment, this complex anionic polymer was converted to a cationic polymer, with a strong affinity to the negatively charged cancer cell membrane, thus facilitating the release of positive CDs-Pt(IV) and effective activation of the drug [171]. Therefore, in order to reduce potential toxic effects and to broaden their application possibilities, surface engineering may be a powerful strategy for the production of functionalized CDs with improved characteristics that could meet the array of particular requirements [172]. Furthermore, a recent study has proven the prooxidant activities of C-dots after exposure to blue light [144]. They reported lipid peroxidation, increased formation of ROS from the electron–hole pair, and demonstrated that the singlet oxygen was generated via both energy-transfer and electron-transfer pathways. However, it was emphasized that CDs’ anti or prooxidant properties depend on light exposure, which is crucial for safe application. A study by Qin et al. has elucidated that C-dots were able to induce apoptosis and autophagy in THP-1-activated macrophages via an elevation in expression levels of caspase 3, caspase 9, Bax, Bad, beclin 1, and LC3-I/II and a decrease in that of Bcl-2 [145]. An excessive generation of ROS was accompanied by a strong inflammatory response delivered via p38MAPK and NF-κB-mediated signaling pathways. This resulted in a considerably augmented expression of proinflammatory cytokines, such as TNF-α, IL-1β, and IL-8.

#### 3.3.4. Nanodiamonds

It has been proposed that NDs appeared to be non-toxic for numerous cell types (immune cell, neurons, and skin cells) and very innoxious regarding the induction of ROS [165]. Moreover, a study conducted on six human cell lines, representatives of vital human organs (kidney, liver, lung, intestine) did not report any significant toxicity, either at the cellular or gene level up to an exposure dose of 250 µg/mL [146]. This is of paramount importance for the huge potential of ND applications in human nanomedicine. In addition, numerous studies have confirmed that the benefit of using nanodiamonds in different model systems far outweigh any adverse effects they may have [147,173,174]. Although a dose-dependent increase in mRNA levels of several cell death and inflammatory markers were reported in the monoblastoid cell line U937 (via TLR4-NF-κB signaling), the same was not observed for SaOS-2 osteoblast-like cells, indicating no evidence of cytotoxicity or inflammation in these cells responsible for bone tissue formation [147]. Moreover, positively charged NDs strongly associate with multiple fibroblast growth factor (FGF) ligands, at very low concentrations, thus mitigating FGF signaling in the targeting cells with no effects on other growth factors’ signaling pathways. This, in return, diminished the pathological FGF signaling seen in cartilage growth in a mouse model [174]. In the Alzheimer’s disease rat model, NDs exhibited protective effects against memory deficit, presumably via modulating NF-kB and STAT3 signaling pathways, consequently inhibiting the pro-inflammatory response (TNF-α and IL-6) and oxidative stress (impeding of iNOS) [174]. However, a study by Dworak et al. has reported a dose-dependent nanodiamond-mediated intracellular redox homeostasis disturbance and ROS generation in human peripheral leukocytes *in vitro*, as well as cell cycle arrest and apoptotic cell death [110].

#### 3.3.5. Carbon Nanotubes

**Figure 5:**
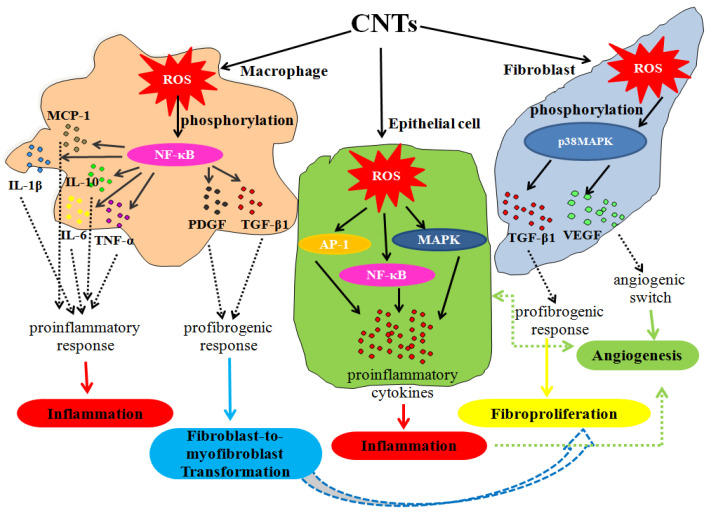
Cell signaling events induced by CNTs, original figure. In different cell types, CNTs induce reactive oxygen species (ROS)-dependent activation of certain cell signaling pathways (NF-κB, MAPK, AP-1), and subsequently, secretion of proinflammatory cytokines (in macrophages and epithelial cells) or profibrogenic and angiogenic factors (in macrophages and lung fibroblasts). Proinflammatory cytokines (IL-1β, IL-6, IL-10, TNF-α, MCP-1) are responsible for inflammation, profibrogenic factors (TGF-β1, PDGF) cause fibroproliferation and differentiation of fibroblast to myofibroblast, while growth factor (VEGF), as well as proinflammatory cytokines, initiates angiogenesis. (NF-nuclear factor, MAPK-mitogen-activated protein kinase, AP-activator protein, IL-interleukine, TNF-tumour necrosis factor, MCP-monocyte chemoattractant protein, TGF-transforming growth factor, PDGF-platelet-derived growth factor, VEGF-vascular endothelial growth factor).

Despite their extraordinary properties and wide application, some research groups have reported possible toxic effects of CNTs and signaling pathways involved in that pathogenesis. The summary of cell signaling pathways activated by CNTs is schematically presented in Figure 5.

Pulmonary cell signaling pathways including NF-κB, STAT-1, MAPK, and RTK lead to proinflammatory cascade and the release of the acute phase cytokines: IL-1β, TNFα, IL-6, and the chemokine IL-8, which are involved in the development of inflammation and fibrosis in lungs after CNTs exposure [136,137]. In addition, it was observed that long CNTs induced activation of the NLRP3 inflammasome via the P2X7 receptor and ROS formation in human primary macrophages [138], thus contributing to the potential health risk. Pacurari et al. have shown that, in a dose-dependent manner, single-walled carbon nanotubes (SWCNTs) in a human mesothelial cell culture induced ROS and, subsequently, the activation of NF-κB, activator protein (AP)-1 and MAPK signaling pathways, thus contributing to the proinflammatory phenotype [139]. Furthermore, ROS-mediated NF-κB activation was responsible for the fibroblast-to-myofibroblast transformation of cultured lung fibroblast after SWCNTs exposure [140]. Accordingly, SWCNT treatment of human lung fibroblasts leads to the activation of p38 MAPK (via ROS phosphorylation) and, subsequently, to the increase in transforming growth factor (TGF)-β1, as well as a vascular endothelial growth factor (VEGF), and thus contributed significantly to the fibroproliferation and angiogenesis in these *in vitro* experimental settings [141]. Similarly, multi-walled carbon nanotubes (MWCNTs) induced ROS-dependent activation of NF-*κ*B in macrophages, thereby inducing the proinflammatory response through TNF-α, IL-1β, IL-6, IL-10, and MCP-1 expression. Likewise, SWCNTs and MWCNTs via NF-κB activation induced synthesis of profibrogenic growth factors TGF-β1 and platelet-derived growth factor (PDGF) from macrophages, further promoting the differentiation of fibroblast to myofibroblast [173]. Moreover, it was reported that besides cytokines augmentation in a dose-dependent manner, MWCNTs could increase the phosphorylation of signaling cascade components of the MAPK/ERK pathway, which is essential for cell cycle and proliferation, cell survival, cell adhesion, etc. [142].

### 3.4. Acute Toxicity of Fullerenol Nanoparticles in Vivo

To the best of our knowledge, investigations concerning the acute toxicity tests of the majority of the above-mentioned carbon nanomaterials are yet to be fully conducted. Due to the fact that, as a scientific group, we have mostly investigated fullerenol nanoparticles (FNPs), in this part, we will briefly focus on their acute toxicity. A few, to date, confirmed adverse effects of FNPs on human, animal, and environmental health are closely related to their physico-chemical properties and low biodegradability at the site of exposure [59,175,176]. In addition, due to the possible different routes of exposure, assessment of acute FNP toxicity is the first step in considering their general toxic effects [17]. Namely, FNP toxicity studies performed mainly in mice and rats [177] were based on the determination of:

- a mean lethal dose (LD_50_) [178,179],
- pharmacological-toxicological profile (i.e., absorption, distribution, metabolism, and excretion (ADME)) [180,181,182,183], as well as
- pathohistological alterations in tissues in which they primarily accumulate [176,180]. In these studies, the acute toxicity of FNPs was directly dependent on the dose administered, the exposure conditions, and the duration of exposure.

**Figure 6:**
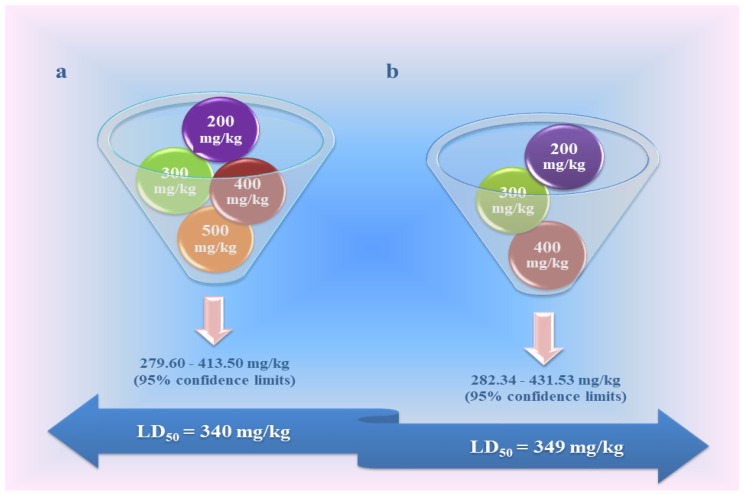
Twenty-four-hour median lethal dose (LD_50_) of fullerenol nanoparticles (FNPs) for an intraperitoneal (i.p.) route of administration; (**a**) calculated LD_50_ value after the application of four increasing doses of FNPs [178]; (**b**) calculated LD_50_ value after the application of three increasing doses of FNPs [179], original figure.

Available studies indicate that FNPs predominantly accumulate in the liver, kidney, and spleen, although they can pass through cell membranes and transport via blood throughout the body [4,180]. It is well-known that FNPs are metabolized in the liver and then eliminated via urine and feces, which initially diminishes their predilection for distribution and toxicity [17]. The relatively lower toxicity has been confirmed in our previous *in vivo* studies when FNPs were applied in an increasing-dose regimen. The calculated medial lethal dose (LD_50_) [178,179], however, has reached similar values, as can be seen in Figure 6a,b.

Accordingly, the first lethal outcomes of rats were registered after a single intraperitoneal administration of FNPs in a dose of 200 mg/kg (2 of 6) in both studies. Upon post-mortem evaluation, severe bleeding from the lungs, heart, and mesentery associated with ascites and pulmonary edema have been observed in fallen animals, especially in rats treated with an FNP dose higher than 200 mg/kg i.p. [179]. The results of other authors show that, in general, the acute toxicity of both fullerenes and their water-soluble fullerenol C_60_(OH)_24_ derivatives is low, while data from studies of repeated toxicity, reproductive toxicity, and carcinogenicity are still insufficient to reach conclusions [184,185]. Thus, after a single dose of 500 mg intraperitoneally administered hydrosoluble fullerene derivative, mice experienced transient weight loss, but without affecting its one-week survival. Ueng and coauthors examined the acute toxicity of fullerenol in mice and obtained an LD_50_ value of 1.2 g/kg i.p. [186]. It should be emphasized that there are a number of different water-soluble fullerenols that have been synthesized and tested. Cai and coworkers showed that fullerenol C_60_(OH)_24_, which we also used, was not only non-toxic when given at 40 mg/kg/day i.p. for 14 consecutive days, but even protected mice from the whole-body exposure to lethal doses of γ-radiation [187].

Taking into consideration the published results of pharmacological and toxicological properties of fullerenol C_60_(OH)_24_ and its structural relative compounds, it is obvious that there is a good basis for its further investigation as a novel promising candidate for the prevention or treatment of different diseases related to inflammation. Namely, FNPs at a dose of 100 mg/kg, which is approximately 1/3 of their LD_50_, produce prominent anti-inflammatory and tissue-protective effects, indicating an excellent safety profile, which is crucial for further preclinical testing [179,188].

## 4. Conclusions

Carbon nanomaterials have found their place in various fields, some of which are electronics, optics, sensorics, and catalysis. Moreover, some of them proved to be potential nanomedicine assets behaving as nanodrug delivery systems, protectors, or bioimaging agents. For this plethora of use, carbon nanomaterials have to thank their physico-chemical properties and chemical functionalization. Despite being promising for the various applications, justified concerns on the impact of carbon nanomaterials have been raised. Research conducted on *in vitro* and *in vivo* models have claimed that some members of the carbon nanomaterial family, besides being genotoxic, also proved to induce oxidative damage, inflammation, and activate different cell signaling pathways that can result in different cellular responses. The size distribution, charge, different synthesis methods, and surface modifications play an essential role in deciding the fate of CNMs in *in vivo* systems. Although enormous research has been put into this issue, the real mechanisms of CNM toxicity are still missing, which is the main obstacle for their clinical promotions. Therefore, further research is needed to address the safety concerns over CNM applications. More *in vitro* studies, including biocompatibility assays, studies on ROS formation, and analyses of inflammation and genotoxicity on numerous healthy and transformed cell lines, will contribute to the essential body of work regarding CNM toxicity. Likewise, *in vivo* testing may further help in designing CNMs with desirable properties and high efficiency. Besides the frequently used rodent models, regular introduction of the zebrafish model into this research may improve the field, as this model has emerged as a reliable and powerful vertebrate model. Finally, all the efforts from *in vitro* and *in vivo* research will contribute to elucidate CNMs’ biological impacts and to translate scientific research into medical practice, subsequently leading to the full potential of CNMs as drug delivery systems, cancer therapeutics, bioimaging systems, gene delivery systems, etc. In order to let the carbon nanomaterials use outweigh their toxicity effects, these powerful materials first ought to be widely assessed for safety in different conditions, doses, and on numerous models.

## Figures and Tables

**Table 1:** The genotoxicity induced by different types of carbon nanomaterials (CNMs).

Type of CNM	Physico-Chemical Characteristics	Cell Line/Animal	Genotoxicity Testing Method	Concentration/Dose	Outcomes	Mechanisms of Genotoxicity	Ref.
**Fullerene**							
C_60_	Particle size: 0.7 nm	A549-Human lung carcinoma cells C57BL/6J or *gpt*delta transgenic male mice	Micronuclei Comet assay	0.02–200 mg/L 0.2 mg of particles per animal	Genotoxic -clastogenic effects DNA damage in the lungs of mice	nc	[108]
C_60_	Particle size: 407–5117 nm	Female rats	8-oxodG, RT-PCR	0.064 or 0.64 mg/kg	Elevated levels of 8-oxodG in the liver and lungs	Indirect (C_60_ generated oxidatively damaged DNA in rodent organs)	[103]
C_60_-Dimethyl sulfoxide (DMSO) suspension	Particle size: 34.95 nm	Adult freshwater fish – *Anabas testudineus*	Micronuclei, Comet assay	5 and 10 mg/L	Genotoxic effects	nc	[106]
C_60_(OH)_24_	-	CHO-K1-Chinese hamster ovary cells	Micronuclei, Chromosomal aberration	12.4–249 mg/L	No genotoxic effects	nc	[104]
C_60_(OH)_24_	Particle size: 180 and 90 nm	Human peripheral blood lymphocytes	Micronuclei, Chromosomal aberration	6.25–249.96 mg/L	No genotoxic effects	nc	[105]
**Nanodiamonds**							
Single-Digit Nanodiamonds	Particle size:50 nm	Insect species – *Achetadomesticus* (*Orthoptera*)	Organism-level end-point (lifespan, body weight, consumption, caloric value of feces, reproduction)	0.02 or 0.2 mg/g dry weight	Genotoxic effect -oxidative damage and feeding disturbances limited to the exposed generation	nc	[109]
Nanodiamond powder	Particle size: <10 nm	Human blood	Micronuclei, FISH, 8-oxoG, Comet assay	1–50 mg/L	Genotoxic effect—elevated level of 8-oxoG at 1 µg/mL, and micronuclei (aneugenic activity) at 10 mg/L, but no induction of DNA double strand breaks	Indirect	[110]
Pristine nanodiamond particles	Particle size: 4–5 nm	C57/BL6-mouse embryonic stem cells	Western blotting-DNA damage and repair biomarkers p53, MOGG-, Rad51, XRCC-4	5 or 100 mg/L	Genotoxic effect–oxidized NDs caused more DNA damage than the pristine/raw NDs	nc	[111]
**Carbon nanotubes**							
SWCNT	Particle size: 0.9–1.7 nm Length: <1 μm	Female rats	8-oxodG	0.064 or 0.64 mg/kg	Genotoxic effect—elevated levels of 8-oxodG in the liver and lung	Indirect (SWCNT generated oxidatively damaged DNA in rodent organs)	[103]
SWCNT	Diameter: 1.1 nm Length: 0.5–100 µm	BEAS 2B-a transformed human bronchial epithelial cell line	Micronuclei, Comet assay	3–360 mg/L	Genotoxic effect	Possibly indirect (contribution by catalyst metals)	[112]
SWCNT	Diameter: 0.4–1.2 nm Length: 1–3 µm	V79 (lung fibroblast line) *S. typhimurium* strains YG1024/YG1029	Comet assay, MN, Ames test	0–9.6 mg/m^2^ 0–0.240 mg/plate	Genotoxic effect -induction of DNA damage at 3 h/960 mg/m^2^and 24 h/≥48 μg/cm^2^; -micronucleus induction at 960 mg/m^2^; -Ames test	nc	[113]
SWCNT	Diameter: 1–4 nm Length: 0.5–1 µm	BEAS-2B-Normal human bronchial epithelial cells	Mitotic spindle analysis, Chromosome number–FISH	0.2–0.8 mg/m^2^ of culture surface area	Genotoxic effect	Direct (association with DNA, mitotic spindle disruption and errors in chromosome number)	[114]
MWCNT	Particle size: 15 ± 5 nm (0.03% Fe, 0% Co, and 0% Ni)	BEAS-2B- human bronchial epithelial cells, SAEC-primary small human airway respiratory epithelial cells	Mitotic spindle analysis, Chromosome number–FISH	0.24–240 mg/m^2^ of culture surface area	Genotoxic effect at 0.24 mg/m^2^ errors in chromosome number and mitotic spindle aberrations	Direct	[115]
MWCNT	Particle size: 5–20 nm Length: 300–2000 nm; Hydrodynamic diameter: 401.3 nm	A549-human lung epithelial cell line	Micronuclei, Western blot (p53)	10 and 50 mg/L	Genotoxic effect at 10 mg/L/24 h	nc	[116]
**Graphene**							
GO	Thickness: 0.7–1.5 nm Mean diameter: 156.4 nm	Mice	Micronuclei	Intravenously 4 mg/kg	Genotoxic effect	Direct (intercalated into DNA) and indirect (inducing ROS)	[35]
	Thickness: 20–30 layers Lateral dimension: <2 µm	Male rats	Comet assay	Inhalation 0.12, 0.47, and 1.88 mg/L	No genotoxic effects	No increased inflammatory markers	[117]
rGO	Particle size: 342 nm Zeta potential: 25 mV Thickness: ∼5 nm	Male rats	Micronuclei	Single tail vein injection of 7 mg/kg, concentration of 1000 mg/L	No genotoxic effects	No inflammatory response	[118]

nc – not considered

**Table 2:** Biological impacts caused by different carbon nanomaterials (CNMs) via various signaling pathways.

Type of CNM	Model System	Receptor/Key Mediator	Signaling Pathways	Biological Impact	Ref.
**Graphene**					
Graphene oxide nanosheets	Macrophage cell RAW264.7	TLR4, TLR9	MyD88, TRAF6, NF-κB	Autophagy of macrophages; Inflammation	[129]
Graphene oxide nanoribbons non-covalently functionalized with PEG-DSPE (O-GNR-PEG-DSPE)	11 different malignant human cell lines	EGFRs	JAK/STAT; MAPK/ERK (Ras/Raf/MEK/ERK)	Cell proliferation	[130]
Printex 90 (Carbon black)	Rat lung epithelial cells	EGFR	MAPK/ERK (Ras/Raf/MEK/ERK)	Cell proliferation	[131,132,133]
		β1-integrin		Cell adhesion; Angiogenesis; Migration	
**Fullerene**					
Fullerenol-1 (13-15 hydroxyl substituents)	A7r5 cells (rat aortic smooth muscle cells, human coronary artery smooth muscle cells	PTK	Protein kinase C	Antiproliferative effect	[134]
Fullerenol 24 hydroxyl substituents)	Human lung cells (type II alveolar epithelial A549)	No data	p38 MAPK	Nrf2-induced antioxidative defense	[135]
**Carbon Nanotubes**					
Long CNT	Met5a mesothelial cells; THP-1 macrophages	TLRs; P2X7	NF-κB; STAT-1; MAPK; RTK	Inflammation and fibrosis in lungs	[136,137,138]
SWCNTs	Human mesothelial cells	EGF; PDGF	NF-κB, AP-1, and MAPK (ERK, p38)	ROS-induced inflammation; Apoptosis	[139]
SWCNTs	Human lung fibroblast (WI-38-VA13)	TGFβ; PDGF	NF-κB	ROS-induced inflammation; Fibroblast-to- myofibroblast transformation	[140]
SWCNTs	Human lung fibroblasts	TGFβ; VEGF	p38 MAPK	Fibroproliferation Angiogenesis	[141]
MWCNT	Mouse macrophages (RAW264.7)	NF-κBp65	NF-*κ*B	Inflammation	[140]
MWCNTs	Human lung fibroblasts (WI38-VA13)	TGFβ; PDGF	NF-κB	Fibroblast-to- myofibroblast transformation	[140]
MWCNTs	Human bronchial epithelial cell line (BEAS–2 B)	ERK1; p38; HSP27	MAPK*/*ERK	Cell proliferation; Cell adhesion	[142]
**Carbon Dots**					
CDs	Yeast cells (*Pichia pastoris)*	No data	No data	ROS response: Growth inhibition	[143]
Pristine CDs (negative charge)	Mouse fibroblasts (NIH/3T3)	No data	No data	ROS response; Arrest of the G2/M phase	[58]
Polyethylenimine-coated CDs (positive charge)	Mouse fibroblasts (NIH/3T3)	No data	No data	Disruption of G0/G1 phase of cell cycle	[58]
Graphene CDs	HUEVEC cells	O_2_	Energy-transfer/Electron-transfer pathways	ROS response	[144]
Graphene CDs	THP-1-activated macrophages	Bcl2, Bax, Bad	p38 MAPK; NF-κB	Apoptosis	[145]
		Beclin 1; LC3		Autophagy	
		NF-κBp65		ROS-induced Inflammation	
**Nanodiamonds**					
NDs	Monoblastoid cells (U937)	TLR4	NF-κB	Apoptosis; Inflammation	[146]
NDs	Alzheimer’s Disease Rat Model	NMDA receptors	NF-κB; STAT3	Neuroprotection—inhibition of Inflammation; Antioxidative defense	[147]
NDs	Human peripheral lymphocytes	O^−^	No data	Apoptosis; Oxidative stress	[110]
